# Neighborhood Disadvantage and Prostate Tumor RNA Expression of Stress-Related Genes

**DOI:** 10.1001/jamanetworkopen.2024.21903

**Published:** 2024-07-12

**Authors:** Joseph Boyle, Jessica Yau, Jimmie L. Slade, Derrick A. Butts, Yuji Zhang, Teklu B. Legesse, Ashley Cellini, Kimberly Clark, Jong Y. Park, Jessica Wimbush, Nicholas Ambulos, Jing Yin, Arif Hussain, Eberechukwu Onukwugha, Cheryl L. Knott, David C. Wheeler, Kathryn Hughes Barry

**Affiliations:** 1 Department of Biostatistics, Virginia Commonwealth University, Richmond; 2 Massey Comprehensive Cancer Center, Richmond, Virginia; 3 Department of Cellular and Molecular Biomedical Science, University of Maryland School of Medicine, Baltimore; 4 Maryland Community Health Engagement Partnership, Upper Marlboro; 5 Prostate Health Matters, Washington, DC; 6 Department of Epidemiology and Public Health, University of Maryland School of Medicine, Baltimore; 7 Program in Oncology, University of Maryland Greenebaum Comprehensive Cancer Center, Baltimore; 8 Department of Pathology, University of Maryland School of Medicine, Baltimore; 9 Pathology Biorepository Shared Service, University of Maryland Greenebaum Comprehensive Cancer Center, Baltimore; 10 Department of Cancer Epidemiology, H. Lee Moffitt Cancer Center, Tampa, Florida; 11 University of Maryland Greenebaum Comprehensive Cancer Center Tumor Registry, Baltimore; 12 Department of Microbiology and Immunology, University of Maryland School of Medicine, Baltimore; 13 Department of Medicine, University of Maryland School of Medicine, Baltimore; 14 Baltimore Veterans Administration Medical Center, Baltimore, Maryland; 15 Department of Practice, Sciences, and Health Outcomes Research, University of Maryland School of Pharmacy, Baltimore; 16 Department of Behavioral and Community Health, University of Maryland, College Park, College Park

## Abstract

**Importance:**

African American men experience greater prostate cancer incidence and mortality than White men. Growing literature supports associations of neighborhood disadvantage, which disproportionately affects African American men, with aggressive prostate cancer; chronic stress and downstream biological impacts (eg, increased inflammation) may contribute to these associations.

**Objective:**

To examine whether several neighborhood disadvantage metrics are associated with prostate tumor RNA expression of stress-related genes.

**Design, Setting, and Participants:**

This cross-sectional study leveraged prostate tumor transcriptomic data for African American and White men with prostate cancer who received radical prostatectomy at the University of Maryland Medical Center between August 1992 and January 2021. Data were analyzed from May 2023 to April 2024.

**Exposures:**

Using addresses at diagnosis, 2 neighborhood deprivation metrics (Area Deprivation Index [ADI] and validated bayesian Neighborhood Deprivation Index) as well as the Racial Isolation Index (RI) and historical redlining were applied to participants’ addresses. Self-reported race was determined using electronic medical records.

**Main Outcomes and Measures:**

A total of 105 stress-related genes were evaluated with each neighborhood metric using linear regression, adjusting for race, age, and year of surgery. Genes in the Conserved Transcriptional Response to Adversity (CTRA) and stress-related signaling genes were included.

**Results:**

A total of 218 men (168 [77%] African American, 50 [23%] White) with a median (IQR) age of 58 (53-63) years were included. African American participants experienced greater neighborhood disadvantage than White participants (median [IQR] ADI, 115 [100-130] vs 92 [83-104]; median [IQR] RI, 0.68 [0.34-0.87] vs 0.11 [0.06-0.14]). ADI was positively associated with expression for 11 genes; *HTR6* (serotonin pathway) remained significant after multiple-comparison adjustment (β = 0.003; SE, 0.001; *P* < .001; Benjamini-Hochberg *q* value = .01). Several genes, including *HTR6*, were associated with multiple metrics. We observed higher expression of 5 proinflammatory genes in the CTRA with greater neighborhood disadvantage (eg, *CXCL8 *and ADI, β = 0.008; SE, 0.003; *P* = .01; *q* value = .21).

**Conclusions and Relevance:**

In this cross-sectional study, the expression of several stress-related genes in prostate tumors was higher among men residing in disadvantaged neighborhoods. This study is one of the first to suggest associations of neighborhood disadvantage with prostate tumor RNA expression. Additional research is needed in larger studies to replicate findings and further investigate interrelationships of neighborhood factors, tumor biology, and aggressive prostate cancer to inform interventions to reduce disparities.

## Key Points

**Question:**

Is neighborhood disadvantage associated with prostate tumor RNA expression of stress-related genes among African American and White men with prostate cancer?

**Findings:**

In this cross-sectional study of 218 men with prostate cancer, neighborhood disadvantage was associated with significantly higher tumor expression of stress-related genes, including several proinflammatory genes. The association between the Area Deprivation Index and *HTR6* (serotonin pathway) remained statistically significant after multiple-comparison adjustment.

**Meaning:**

These findings suggest that neighborhood disadvantage is associated with higher expression of stress-related genes, which may contribute to an increased risk of aggressive prostate cancer and warrants follow-up in future studies.

## Teaser

This cross-sectional study evaluates whether there is an association between neighborhood disadvantage and the tumor RNA expression of stress-related genes in African American and White men with prostate cancer.

## Introduction

Prostate cancer is the leading cause of cancer incidence and second leading cause of cancer mortality among men in the United States.^1^ Prostate cancer rates are relatively high among African American men, with an incidence rate more than 1.5 times and a mortality rate more than 2 times that of White men. African American men are more likely to be diagnosed with advanced prostate cancer, partly due to reduced access to health care and screening.^2,3^ Additionally, African American men are thought to be more likely to develop aggressive prostate cancer, which is more likely to be fatal, but the reasons are unclear.^3^

Increasing literature supports that neighborhood disadvantage (eg, neighborhood socioeconomic deprivation), which disproportionately affects African American individuals,^4,5^ is associated with a higher likelihood of advanced or aggressive prostate cancer^5,6,7,8^ and therefore may contribute importantly to disparities. The downward causal model from the University of Chicago posits that population-level (eg, neighborhood) factors have downstream biological impacts that influence disease development, for example via increased allostatic load due to chronic stress.^9,10^ Under this framework, we and others hypothesize that neighborhood disadvantage may increase the development of aggressive prostate cancer in part through chronic stress and related downstream biological effects including increased inflammation, which has been linked with prostate cancer risk and progression in previous studies.^11,12,13,14,15^ Notably, previous research has also suggested associations between neighborhood disadvantage and inflammation-related genes in serum.^16^

There is also evidence that neighborhood-level effects of historical structural racism (eg, residential racial segregation and redlining) influence health, including cancer outcomes, in the present day. Residential racial segregation refers to the tendency for members of similar racial groups to inhabit the same neighborhoods, which may bring about a concentration of disadvantage, depending on the racial group and economics. Redlining refers to a concerted effort by the US government to systematically deny mortgage applications or refinancing in certain neighborhoods, often based on race.^17,18,19^ Residence in historically redlined neighborhoods has been linked to elevated risk of late-stage lung cancer among men, even if such neighborhoods became less deprived over time,^20^ and to elevated hazards for breast cancer–specific and all-cause mortality in women.^21^

We hypothesized that neighborhood disadvantage would affect the expression of stress-related genes, which in turn would contribute to an increased risk of aggressive prostate cancer. In the present study, we assessed associations of 4 neighborhood disadvantage metrics with prostate tumor RNA expression of stress-related genes among African American and White men with prostate cancer. We included 2 neighborhood deprivation metrics, the publicly available Area Deprivation Index (ADI)^22,23^ and a validated Neighborhood Deprivation Index (NDI) developed by our group,^24,25^ which was defined using a flexible bayesian index model that estimates importance weights for model components and allows the model to be tailored to the outcome. We also included a measure of racial residential segregation known as the Racial Isolation Index (RI),^26^ as well as historical redlining. It is critical to investigate the association of different neighborhood factors with prostate tumor biology to help understand how these factors may contribute to the development of aggressive prostate cancer and related disparities.

## Methods

### Study Population

Our cross-sectional study included 218 men with prostate cancer (168 [77%] African American men and 50 [23%] White men) who received radical prostatectomy surgery at the University of Maryland Medical Center from August 1992 to January 2021, had available RNA expression data from prostate tumor tissue, and had a valid residential, noninstitutional address at the time of diagnosis available for geocoding; African American men were overselected (see sample selection flowchart in eFigure 1 in Supplement 1). We included self-reported race (via electronic medical records) based on relevance of race as a social construct to our hypothesis that neighborhood and social factors may affect stress-related pathways. We selected men who received surgery to ensure that there would be adequate tissue for downstream molecular assays and due to concern that biopsy samples may not reflect the overall tumor. The current study was approved by the institutional review board (IRB) at the University of Maryland, Baltimore, which was the IRB of record for this project, and followed the Strengthening the Reporting of Observational Studies in Epidemiology (STROBE) reporting guidelines. We obtained a waiver of informed consent as part of the approved IRB protocol for this study.

### Study Outcome: RNA Expression

We leveraged transcriptomic data from formalin-fixed, paraffin-embedded (FFPE) prostate tumor tissue from our previous studies. The pathology and RNA extraction approaches have been described previously^27^ (also see more detail in eMethods in Supplement 1). Briefly, RNA was extracted from prostate tumor samples using the RNEasy FFPE kit (Qiagen). We obtained RNA expression data from the Human Clariom D array, which measures expression for 138 745 transcript clusters (TCs). We analyzed a subset of 105 TCs corresponding to stress-related genes, including those in the Conserved Transcriptional Response to Adversity (CTRA)^28^ and stress-related signaling pathways, encompassing the adrenergic, glucocorticoid, dopaminergic, serotoninergic, and muscarinic systems^29^ (eTable 1 in Supplement 1). Various stress-related signaling genes have been previously linked with lethal prostate cancer^29^ and there is relevance of CTRA genes (eg, proinflammatory genes) to prostate cancer risk and progression.^11,12,13^

### Neighborhood Measures

We geocoded the residential address at diagnosis for each participant using ArcGIS (Esri), which is a required field within the University of Maryland Greenebaum Comprehensive Cancer Center tumor registry. We included several measures of neighborhood disadvantage (more detail appears in eMethods in Supplement 1), considering the census tract containing each participant’s address at diagnosis as the neighborhood. The first measure was the publicly available ADI, which includes 17 neighborhood-level variables in the income, education, employment, and housing quality domains and is available for 2005 and later.^22,23^ Greater values of the ADI indicate greater deprivation. We calculated the ADI measure for 160 participants (117 African American and 43 White).

Our second measure was a custom NDI that we fit with the validated bayesian index model.^24^ The construction of the NDI was similar to that of the ADI, but importance weights for index components were estimated in modeling, allowing identification of the most important variables for the given outcome. Greater values corresponded to greater deprivation. We calculated the NDI measure for 211 participants (162 African American and 49 White).

Our third measure was the spatial RI index,^26^ which was available for 2009 and later. This measure ranged from 0 to 1, with higher values corresponding to higher African American residential segregation. We calculated the RI measure for 136 participants (99 African American and 37 White).

Our fourth measure was historical redlining. We used the Home Owners’ Loan Corporation (HOLC) redlining map of the Baltimore area from the 1930s. We categorized redlining as a binary variable (lowest grade, D, compared with any other grade [A, B, C, or no grade]). We used the digitized redlining map from the Mapping Inequality project.^30^ We calculated the redlining measure for 218 participants (168 African American and 50 White).

### Statistical Analysis

We fit linear regression models for each gene in relation to each neighborhood measure, adjusting for race, age at surgery, and year of surgery (more detail appears in eMethods in Supplement 1). For the ADI, RI, and historical redlining exposures, we fit frequentist linear regression models for the log base-2 transformed gene expression values. We reported coefficients, standard errors, *P* values, and *q* values (*P* values adjusted for 105 genes tested with each exposure with the Benjamini-Hochberg method^31^), with a conventional .05 level of significance. For the NDI, we fit bayesian linear regression models, reporting coefficients, credible intervals, and exceedance probabilities (EPs). We calculated Spearman correlations between the continuous neighborhood measures to evaluate their agreement. As a sensitivity analysis, we repeated the adjusted models among African American men only to assess whether the significant findings were similar to the primary analysis. We also assessed characteristics of the larger group of men receiving radical prostatectomy for prostate cancer at the University of Maryland Medical Center from 1992 to 2021 to assess the extent to which our sample reflected this larger group. We performed an additional sensitivity analysis comparing socioeconomic and health care access indicators^32,33^ between geographic areas in Maryland (census tracts, counties) contributing to our sample vs those that did not to explore the representativeness of our sample with respect to Maryland as a whole. Finally, we assessed whether primary payer at diagnosis (proxy for individual-level socioeconomic status) confounded any top associations (ie, those with *P* < .05) in the primary analysis. We performed analyses in R version 4.3.1 (R Project for Statistical Computing); tests were 2-tailed. Statistical analysis was conducted from May 2023 to April 2024.

## Results

Most of the sample comprised African American men (168 [77%], with 50 [23%] White men), and the median (IQR) age at surgery was 58 (53-63) years (Table 1). Approximately 21% of the men (46 men) had an established family history of prostate cancer. Most men had a pathologic tumor stage of 2 or 3 (163 of 165 men with available data [98%]). The most common Gleason pattern was 3 + 4 (101 men [46%]); most participants (150 [69%]) had intermediate-risk prostate cancer per American Urological Association definitions. A small population of the men (8 men [6%]) had known nodal involvement (pN1). Approximately 93% of the men underwent surgery within 1 year of diagnosis (202 men). Aside from a larger proportion of African American men, study participant characteristics were generally similar to those of the broader group of African American and White men receiving radical prostatectomy for prostate cancer at the University of Maryland Medical Center. Specifically, among the 730 men meeting these criteria, the mean (SD) age at surgery was 58.9 (6.6) years, and 295 (40%) were African American; among the 659 men with available pathologic stage information, the distribution was: stage 1, 43 (7%); stage 2, 433 (66%); stage 3, 156 (24%); stage 4, 27 (4%). The continuous neighborhood measures were moderately to highly correlated, with correlations of 0.93 for ADI and NDI, 0.70 for ADI and RI, and 0.69 for NDI and RI. We found that Maryland areas (counties or census tracts) contributing participants to our study did not systematically differ from those that did not contribute with respect to socioeconomic indicators or access to health care (eTable 2 in Supplement 1).

**Table 1.** Study Population Characteristics for African American and White Men Who Received Radical Prostatectomy Surgery for Prostate Cancer at the University of Maryland Medical Center, 1992-2021

Characteristic	Participants, No. (%) (N = 218)^a^
Race	
African American	168 (77)
White	50 (23)
Age at surgery, median (IQR), y	58 (53-63)
Year of surgery	
1992-1997	23 (11)
1998-2004	30 (14)
2005-2010	44 (20)
2011-2016	74 (34)
2017-2021	47 (22)
Family history of prostate cancer	
Yes	46 (21)
No	101 (46)
Unknown	71 (33)
BMI, median (IQR)	27.7 (25.0-31.5)
Preoperative PSA, ng/mL	6.3 (4.9-9.5)
Pathologic tumor stage	
1	1 (1)
2	108 (65)
3	55 (33)
4	1 (1)
Pathologic nodal involvement	
N0	125 (94)
N1	8 (6)
Pathologic Gleason score or pattern	
Total Gleason score ≤6	72 (33)
3 + 4	101 (46)
4 + 3 or Total Gleason score ≥8	45 (21)
Prostate cancer risk stratification	
Low-risk	46 (21)
Intermediate-risk	150 (69)
High-risk	19 (9)
Indeterminate	3 (1)

Abbreviations: BMI, body mass index (calculated as weight in kilograms divided by height in meters squared); PSA, prostate-specific antigen.

SI conversion factor: To convert PSA to micrograms per liter, multiply by 1.

^a^ Some percentages may not sum exactly to 100 due to rounding. There were 28 missing values for BMI and 50 missing values for preoperative PSA. Count and percentage for pathologic tumor stage and pathologic nodal involvement are calculated for observations without missing information (unknown status for 53 and 85 participants for these variables, respectively, predominantly for earlier cases when this information was not required to be collected by the tumor registry). Risk stratification was based on guidelines from the American Urological Association; pathologic tumor stage and grade and preoperative PSA information was used (see eMethods in Supplement 1 for details).

**Figure 1.**
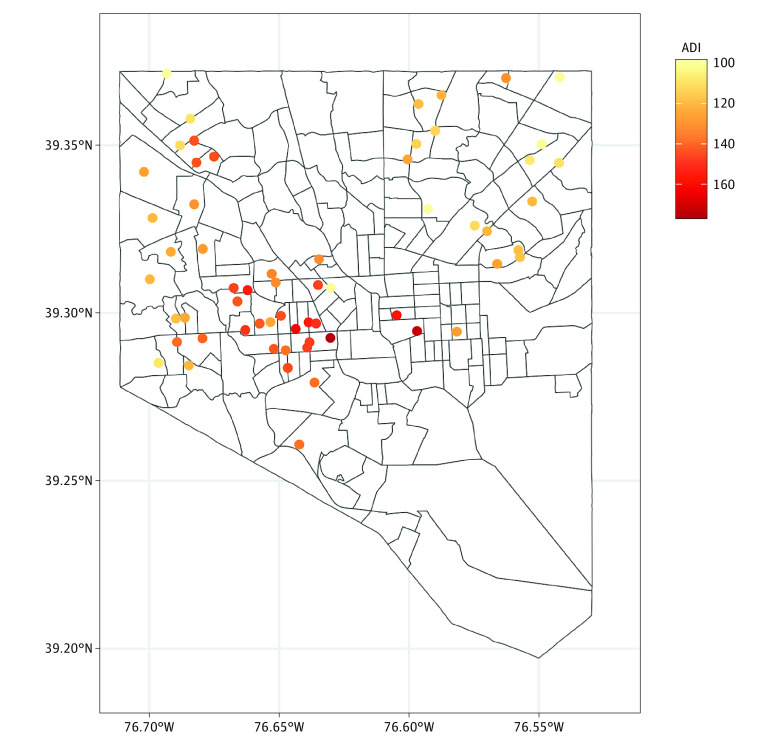
Area Deprivation Index (ADI) Values of Participants in the Baltimore City Area Higher values indicate greater neighborhood deprivation. Participant locations are slightly jittered to maintain confidentiality.

Values of the ADI varied across the state of Maryland, with higher deprivation generally occurring in Baltimore compared with the rest of the state (eFigure 2 in Supplement 1). Within Baltimore, the greatest ADI values were concentrated in central and western neighborhoods of the city (Figure 1). Median (IQR) values for the ADI were 115 (100-130) for African American men and 92 (83-104) for White men, indicating greater neighborhood deprivation in our sample for African American men. Eleven genes were associated with ADI (Table 2). For each of these genes, greater deprivation was associated with greater expression. Notably, 1 gene (*HTR6* in the serotonin pathway) remained significantly associated with ADI after adjustment for multiple comparisons (β = 0.003; SE, 0.001; *P* < .001; *q* value = .01).

**Table 2.** Summary of Stress-Related Genes Associated With the Neighborhood Measures in Adjusted Models Among African American and White Men Who Received Radical Prostatectomy Surgery for Prostate Cancer at the University of Maryland Medical Center, 1992-2021

TC	Gene	Pathway	Beta (SE)^a^	*P* value^a^	q Value^b^
**ADI, 160 participants**					
TC0100007175.hg.1	*HTR6*	Serotonin	0.003 (0.001)	<.001	.01
TC0500012405.hg.1	*HTR4*	Serotonin	0.004 (0.001)	.003	.15
TC0300009685.hg.1	*HTR3D*	Serotonin	0.003 (0.001)	.006	.18
TC1000008401.hg.1	*IFIT5*	Type I IFN responses	0.005 (0.002)	.009	.18
TC1000008396.hg.1	*IFIT2*	Type I IFN responses	0.005 (0.002)	.009	.18
TC0400007836.hg.1	*CXCL8*	Proinflammatory	0.008 (0.003)	.01	.21
TC0200007096.hg.1	*FOSL2*	Proinflammatory	0.004 (0.002)	.02	.30
TC0200013916.hg.1	*IL1B*	Proinflammatory	0.005 (0.002)	.02	.30
TC2100007205.hg.1	*MX2*	Type I IFN responses	0.004 (0.002)	.03	.33
TC0100008815.hg.1	*IFI44L*	Type I IFN responses	0.005 (0.003)	.04	.39
TC1400007706.hg.1	*FOS*	Proinflammatory	0.010 (0.005)	.05	.43
**RI; 136 participants**					
TC0100007175.hg.1	*HTR6*	Serotonin	0.179 (0.063)	.005	.54
TC2200008174.hg.1	*IGLL1*	Antibody synthesis	0.231 (0.097)	.02	.63
TC0300009686.hg.1	*HTR3C*	Serotonin	0.324 (0.143)	.03	.63
TC1000008396.hg.1	*IFIT2*	Type I IFN responses	0.342 (0.152)	.03	.63
TC2100007205.hg.1	*MX2*	Type I IFN responses	0.318 (0.153)	.04	.63
TC1100008123.hg.1	*ADRBK1*	Adrenergic	0.196 (0.095)	.04	.63
TC1600008712.hg.1	*IRF8*	Type I IFN responses	0.327 (0.159)	.04	.63
**Redlining; 218 participants**					
TC0900008660.hg.1	*PTGS1*	Proinflammatory	0.337 (0.133)	.01	.59
TC2100007205.hg.1	*MX2*	Type I IFN responses	0.288 (0.115)	.01	.59
TC1000008396.hg.1	*IFIT2*	Type I IFN responses	0.266 (0.116)	.02	.59
TC1000008968.hg.1	*ADRB1*	Adrenergic	−0.352 (0.154)	.02	.59
TC0100018367.hg.1	*CHRM3*	Muscarinic	0.332 (0.153)	.03	.59
TC1200008920.hg.1	*OAS3*	Type I IFN responses	0.185 (0.087)	.03	.59
**NDI; 211 participants**					
TC1400007706.hg.1	*FOS*	Proinflammatory	0.195 (95% CrI, −0.045 to 0.404)	EP, 95.8%^c^	NA

Abbreviations: ADI, Area Deprivation Index; CrI, credible interval; EP, exceedance probability; IFN, interferon; NA, not applicable; NDI, Neighborhood Deprivation Index; RI, Racial Isolation Index; TC, transcript cluster.

^a^ From linear regression modeling the given gene association with the neighborhood measure, adjusted for race, age at surgery, and year of surgery.

^b^ *P* value adjusted for multiple comparisons using the Benjamini-Hochberg false-discovery rate approach.

^c^ EP indicates the probability of being greater than zero.

One gene (*FOS*, a proinflammatory gene in the CTRA) was associated with the NDI estimated by the bayesian index model (Table 2), with an exceedance probability of 95.8%, indicating significance. The most important variables estimated in the NDI for *FOS* were percentage in tract without a high school diploma and percentage of structures in tract that were vacant (weights of 21.9% and 23.9%).

**Figure 2.**
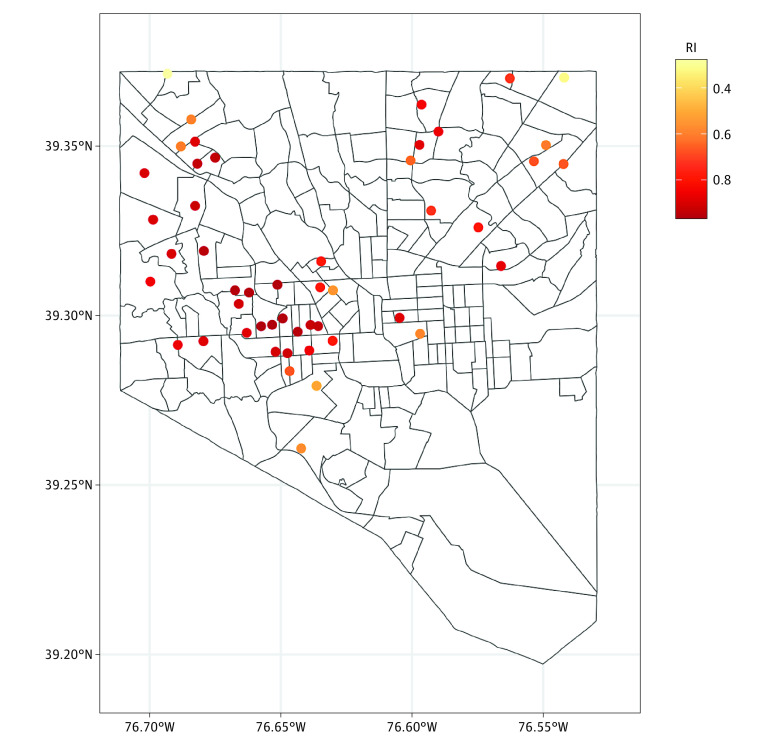
Racial Isolation Index (RI) Values of Participants in the Baltimore City Area Higher values indicate greater African American residential segregation. Participant locations are slightly jittered to maintain confidentiality.

African American racial isolation measured by the RI index also varied, with larger RI values occurring within the Baltimore area than outside it (eFigure 3 in Supplement 1) and particularly in central and western neighborhoods in Baltimore (Figure 2). The median (IQR) values for the RI were 0.68 (0.34-0.87) for African American men and 0.11 (0.06-0.14) for White men, indicating that African American men in our sample tended to reside in areas with higher African American residential segregation. Seven genes were associated with RI (Table 2), although none of these findings persisted after multiple-comparison adjustment. Similar to the top ADI findings, the top genes associated with RI showed positive associations with higher disadvantage.

**Figure 3.**
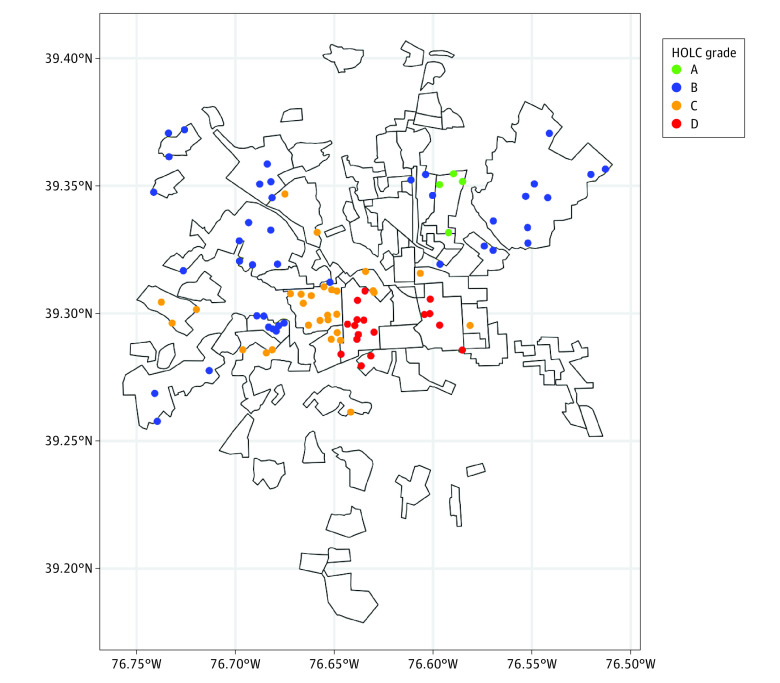
Neighborhood Grades From 1930s Home Owners’ Loan Corporation Map for Participants in the Baltimore Area All neighborhoods receiving grades are plotted on the map, and the residential locations of participants in the Baltimore area residing in these neighborhoods are overlaid. Participant locations are slightly jittered to maintain confidentiality.

Sixteen participants in our sample lived in a census tract that formerly received the lowest grade (D) from HOLC (Figure 3). Notably, neighborhoods receiving the lowest grade in the 1930s in the central and western areas of Baltimore had a large degree of neighborhood deprivation as measured by the ADI and RI many decades later (comparing Figure 3 with Figures 1 and 2). Six genes were associated with living in a formerly redlined neighborhood (Table 2), and the expression of all but 1 gene (*ADRB1*) was positively associated with the redlined status, although none of these findings remained significant after multiple-comparison adjustment.

Some of the genes were associated with more than 1 neighborhood measure at the *P* < .05 level. *HTR6* was associated with both ADI and RI. *FOS* was associated with both ADI and NDI. *IFIT2* and *MX2* (type I interferon responses) were associated with ADI, RI, and historical redlining. Of note, there were several proinflammatory genes that were associated with neighborhood disadvantage at the *P* < .05 level (eg, *CXCL8 *and ADI, β = 0.008; SE, 0.003; *P* = .01; *q* value = .21; *IL1B* and *FOSL2* also showed higher expression with higher ADI; *FOS* with ADI and NDI; and *PTGS1* with redlining). However, the results were not statistically significant after adjustment for multiple comparisons.

The top findings for the sensitivity analysis among African American men only (representing the majority of the study population) were similar to the results from the primary analysis among African American and White men combined (eTable 3 in Supplement 1). Additionally, we found that insurance status (primary payer at diagnosis) was not an important confounder of our top associations between the neighborhood metrics and prostate tumor RNA expression of stress-related genes, with relatively small percentage changes in the coefficient for the neighborhood measures after adjustment for this variable (eTable 4 in Supplement 1).

## Discussion

In this study, we evaluated neighborhood disadvantage (based on ADI, NDI, RI, and historical redlining) and prostate tumor RNA expression of stress-related genes among African American and White men. To our knowledge, our study is one of the first to identify associations between neighborhood factors and RNA expression in prostate tumor tissue. We identified several associations at the *P* < .05 level between neighborhood factors and stress-related genes. For most of the top findings, including 5 proinflammatory genes in the CTRA, higher disadvantage was associated with higher RNA expression. Several of the top genes, including *HTR6* (serotonin pathway) were associated with more than 1 neighborhood measure. Notably, the association between ADI and *HTR6* remained statistically significant after multiple-comparison adjustment.

*HTR6*, which was positively associated with both ADI and RI, encodes a receptor that is part of the 7-transmembrane G protein–coupled receptor family and plays a role in activating the cyclic AMP-dependent signaling pathway. This receptor is hypothesized to be involved in regulating cholinergic neuronal transmission. Among other roles, the cholinergic system is important in regulating immune response, and its dysregulation is thought to contribute to some inflammatory and autoimmune conditions.^34^ There is also evidence that expression of *HTR* genes may be associated with lethal prostate cancer. A previous study^29^ reported mixed associations between prostate tumor expression of 17 *HTR *genes and lethal prostate cancer. Among these, *HTR2A* and *HTR2B* were positively associated with lethal prostate cancer, while *HTR6* was inversely associated. It is unclear why the direction of the *HTR6* association differs from that we observed for *HTR6* and neighborhood disadvantage, but this may be due in part to different stage distributions between the studies, which could have influenced expression levels and warrants further investigation in future studies.

It is noteworthy that we observed positive associations between neighborhood disadvantage and several proinflammatory genes given that these genes tend to be upregulated in the CTRA.^28^ A recent prostate cancer case-control study^16^ found that neighborhood deprivation was associated with increased activity scores for serum proteome-defined pathways related to inflammation among control participants. Moreover, inflammation has been linked with increased prostate tumor development and progression.^11,12,13^ Therefore, findings for these genes were in an expected direction.

Findings from our study support and build on existing literature on the emerging importance of neighborhood factors for aggressive prostate cancer and prostate cancer disparities. Similar to previous studies,^4,5^ we found that African American men with prostate cancer were more likely than their White counterparts to reside in disadvantaged neighborhoods. Various studies have observed associations between neighborhood disadvantage and advanced or aggressive prostate cancer.^5,6,7,8^ Moreover, a study in Detroit^35^ found that neighborhood socioeconomic status was an important factor contributing to racial disparities in prostate cancer mortality. We recognize there are many factors that may contribute to these associations.^36^ Here we investigated one biological pathway in relation to the neighborhood factors based on accumulating evidence for a role of chronic stress in associations for neighborhood factors and aggressive tumor biology.^15^

### Strengths and Limitations

Our study has several strengths. First, we used several measures of neighborhood disadvantage, which provided a more comprehensive view than would have been allowed by analyzing only 1 factor and included many components of disadvantage. A second strength was the use of the flexible, validated bayesian index regression model to compute the NDI. This model has shown improved goodness of fit when compared with other methods, such as principal component analysis and factor analysis, which can result in indices that are more difficult to interpret and do not consider the relative importance of individual model components for the outcome.^37,38^ In our study, *FOS* was associated with both ADI and NDI, but we found that the association was largely driven by 2 NDI components. A third strength was the availability of transcriptomic data, which allowed evaluation of a number of stress-related genes.

Study limitations included the relatively small sample sizes for our analyses (ranging from 136 to 218 based on data availability for different disadvantage metrics in different time periods) and selection of men undergoing surgery. Therefore, our findings may not be generalizable to a broader group of men with prostate cancer. However, we found that areas contributing participants to our sample did not systematically differ from other areas in Maryland with respect to socioeconomic indicators or health care access factors, reducing the likelihood of selection bias. Second, we defined the measures of neighborhood disadvantage based on each participant’s residence at diagnosis, and we did not have access to their residential histories. This could have resulted in some exposure misclassification. Third, we had limited data on individual-level factors (eg, income) that could be contributing to our findings as confounders, effect modifiers, or mediators.^39,40,41^ However, we found that primary payer at diagnosis (which we expect to be associated with individual socioeconomic status) was not an important confounder of the observed associations.

## Conclusions

In conclusion, we identified several suggestive associations between neighborhood disadvantage metrics and prostate tumor RNA expression of stress-related genes among African American and White men with prostate cancer, which warrant follow-up in larger studies. These findings support a potential link between neighborhood factors and stress-related pathways, which may in turn contribute to an increased risk of aggressive prostate cancer. Additional research is needed to further investigate the interrelationships of neighborhood factors, individual factors, prostate tumor biology, tumor aggressiveness, and prostate cancer outcomes, including mediation analyses with survival outcomes, to help inform interventions that will reduce prostate cancer disparities.

## Notes

Supplement 1. **eTable 1. **List of Stress-Related Genes Evaluated in the Present Study and Their Associated Pathways **eTable 2.** Summary of Comparing Socioeconomic and Health Care Access Indicators Between Counties and Census Tracts in Maryland Contributing vs Not Contributing Participants to Our Sample of Men Who Received Radical Prostatectomy Surgery for Prostate Cancer at the University of Maryland Medical Center, 1992-2021 **eTable 3.** Summary of Genes Associated With the Neighborhood Measures in Adjusted Models for African American Men Who Received Radical Prostatectomy Surgery for Prostate Cancer at the University of Maryland Medical Center, 1992-2021 **eTable 4.** Summary of Sensitivity Analysis Additionally Adjusting for Primary Payer at Diagnosis for Genes Associated With the Neighborhood Measures in Adjusted Models Among African American and White Men Who Received Radical Prostatectomy Surgery for Prostate Cancer at the University of Maryland Medical Center, 1992-2021 **eFigure 1.** Flowchart Illustrating Sample Selection **eFigure 2.** Area Deprivation Index Values of Participants in Sample **eFigure 3.** Racial Isolation Index Values of Participants in Sample
eMethods.

eReferences.

Supplement 2.
Data Sharing Statement
