# Supplementary material for: Neighborhood Disadvantage and Prostate Tumor RNA Expression of Stress-Related Genes
Source: JAMA Netw Open. 2024 Jul 12;7(7):e2421903. doi: 10.1001/jamanetworkopen.2024.21903 (PMC11245728; doi:10.1001/jamanetworkopen.2024.21903)
Supplement: Supplement 1. — eTable 1. List of Stress-Related Genes Evaluated in the Present Study and Their Associated Pathways eTable 2. Summary of Comparing Socioeconomic and Health Care Access Indicators Between Counties and Census Tracts in Maryland Contributing vs Not Contributing Participants to Our Sample of Men Who Received Radical Prostatectomy Surgery for Prostate Cancer at the University of Maryland Medical Center, 1992-2021 eTable 3. Summary of Genes Associated With the Neighborhood Measures in Adjusted Models for African American Men Who Received Radical Prostatectomy Surgery for Prostate Cancer at the University of Maryland Medical Center, 1992-2021 eTable 4. Summary of Sensitivity Analysis Additionally Adjusting for Primary Payer at Diagnosis for Genes Associated With the Neighborhood Measures in Adjusted Models Among African American and White Men Who Received Radical Prostatectomy Surgery for Prostate Cancer at the University of Maryland Medical Center, 1992-2021 eFigure 1. Flowchart Illustrating Sample Selection eFigure 2. Area Deprivation Index Values of Participants in Sample eFigure 3. Racial Isolation Index Values of Participants in Sample eMethods. eReferences. [file jamanetwopen-e2421903-s001.pdf]

Supplemental Online Content

Boyle J, Yau J, Slade JL, et al. Neighborhood disadvantage and prostate tumor RNA expression of stress-related genes. *JAMA Netw Open.* 2024;7(7):e2421903. doi:10.1001/jamanetworkopen.2024.21903

eTable 1. List of Stress-Related Genes Evaluated in the Present Study and Their Associated Pathways

eTable 2. Summary of Comparing Socioeconomic and Health Care Access Indicators Between Counties and Census Tracts in Maryland Contributing vs Not Contributing Participants to our Sample of Men Who Received Radical Prostatectomy Surgery for Prostate Cancer at the University of Maryland Medical Center, 1992-2021

eTable 3. Summary of Genes Associated With the Neighborhood Measures in Adjusted Models for African American Men Who Received Radical Prostatectomy Surgery for Prostate Cancer at the University of Maryland Medical Center, 1992-2021

eTable 4. Summary of Sensitivity Analysis Additionally Adjusting for Primary Payer at Diagnosis for Genes Associated With the Neighborhood Measures in Adjusted Models Among African American and White Men Who Received Radical Prostatectomy Surgery for Prostate Cancer at the University of Maryland Medical Center, 1992-2021

eFigure 1. Flowchart Illustrating Sample Selection

eFigure 2. Area Deprivation Index Values of Participants in Sample

eFigure 3. Racial Isolation Index Values of Participants in Sample

eMethods.

eReferences.

This supplemental material has been provided by the authors to give readers additional information about their work.

© 2024 Boyle J et al. *JAMA Network Open.*

eTable 1. List of Stress-Related Genes Evaluated in the Present Study and Their Associated Pathways

Gene (alphabetical order)	TC	Pathway
**Stress-related genes in the Conserved Transcriptional Response to Adversity (Fredrickson et al, *PNAS* 2013)**		
CXCL8^	TC0400007836.hg.1	Proinflammatory
FOS	TC1400007706.hg.1	Proinflammatory
FOSB	TC1900008326.hg.1	Proinflammatory
FOSL1	TC1100011259.hg.1	Proinflammatory
FOSL2	TC0200007096.hg.1	Proinflammatory
GBP1	TC0100014852.hg.1	Type I IFN responses
IFI16	TC0100010244.hg.1	Type I IFN responses
IFI27	TC1400008056.hg.1	Type I IFN responses
IFI27L1	TC1400008055.hg.1	Type I IFN responses
IFI27L2	TC1400010041.hg.1	Type I IFN responses
IFI30; PIK3R2‡	TC1900007365.hg.1	Type I IFN responses
IFI35	TC1700007931.hg.1	Type I IFN responses
IFI44	TC0100008816.hg.1	Type I IFN responses
IFI44L	TC0100008815.hg.1	Type I IFN responses
IFI6	TC0100013445.hg.1	Type I IFN responses
IFIH1	TC0200014772.hg.1	Type I IFN responses
IFIT1	TC1000008400.hg.1	Type I IFN responses
IFIT1B^	TC1000008399.hg.1	Type I IFN responses
IFIT2	TC1000008396.hg.1	Type I IFN responses
IFIT3	TC1000008397.hg.1	Type I IFN responses
IFIT5	TC1000008401.hg.1	Type I IFN responses
IFITM1	TC1100012949.hg.1	Type I IFN responses
IFITM2	TC1100012948.hg.1	Type I IFN responses
IFITM3	TC1100009657.hg.1	Type I IFN responses
IFITM4P	TC0600011324.hg.1	Type I IFN responses
IFITM5	TC1100013119.hg.1	Type I IFN responses
IFNB1	TC0900009688.hg.1	Type I IFN responses
IGLL1	TC2200008174.hg.1	Antibody Synthesis
IGLL3P^	TC2200006920.hg.1	Antibody Synthesis
IL1A	TC0200013913.hg.1	Proinflammatory
IL1B	TC0200013916.hg.1	Proinflammatory
IL6	TC0700006890.hg.1	Proinflammatory
IRF2	TC0400012606.hg.1	Type I IFN responses
IRF7	TC1100009685.hg.1	Type I IFN responses
IRF8	TC1600008712.hg.1	Type I IFN responses
JUN	TC0100014349.hg.1	Proinflammatory
JUNB	TC1900007096.hg.1	Proinflammatory
JUND	TC1900010009.hg.1	Proinflammatory

© 2024 Boyle J et al. *JAMA Network Open.*

MX1	TC2100007208.hg.1	Type I IFN responses
MX2	TC2100007205.hg.1	Type I IFN responses
NFKB1	TC0400008271.hg.1	Proinflammatory
NFKB2	TC1000008727.hg.1	Proinflammatory
OAS1	TC1200012708.hg.1	Type I IFN responses
OAS2	TC1200008921.hg.1	Type I IFN responses
OAS3	TC1200008920.hg.1	Type I IFN responses
OASL	TC1200012190.hg.1	Type I IFN responses
PTGS1	TC0900008660.hg.1	Proinflammatory
PTGS2	TC0100016715.hg.1	Proinflammatory
REL	TC0200016452.hg.1	Proinflammatory
RELA	TC1100011243.hg.1	Proinflammatory
RELB	TC1900008300.hg.1	Proinflammatory
TNF	TC0600007596.hg.1	Proinflammatory
**Stress-related signaling genes (Lu et al, *Clin Cancer Res* 2016)**		
ADRA1A	TC0800012393.hg.1	Adrenergic
ADRA1B	TC0500009264.hg.1	Adrenergic
ADRA1D	TC2000008253.hg.1	Adrenergic
ADRA2A	TC1000008913.hg.1	Adrenergic
ADRA2B	TC0200013509.hg.1	Adrenergic
ADRA2C	TC0400006616.hg.1	Adrenergic
ADRB1	TC1000008968.hg.1	Adrenergic
ADRB2	TC0500009046.hg.1	Adrenergic
ADRB3	TC0800010144.hg.1	Adrenergic
ADRBK1	TC1100008123.hg.1	Adrenergic
ADRBK2	TC2200006935.hg.1	Adrenergic
CALY	TC1000012395.hg.1	Dopamine
CHRM1	TC1100011110.hg.1	Muscarinic
CHRM2	TC0700009277.hg.1	Muscarinic
CHRM3	TC0100018367.hg.1	Muscarinic
CHRM3	TC0100018366.hg.1	Muscarinic
CHRM4	TC1100010703.hg.1	Muscarinic
CHRM5	TC1500006811.hg.1	Muscarinic
COX2	TC0M00006437.hg.1	Proinflammatory
DRD1	TC0500012909.hg.1	Dopamine
DRD2	TC1100012350.hg.1	Dopamine
DRD3	TC0300012043.hg.1	Dopamine
DRD4	TC1100006481.hg.1	Dopamine
DRD5	TC0400006853.hg.1	Dopamine
FKBP4	TC1200006503.hg.1	Glucocorticoid
HSP90AA1	TC1400010257.hg.1	Glucocorticoid
HSP90AB1	TC0600008129.hg.1	Glucocorticoid
HSPA1A; HSPA1B	TC0600007613.hg.1	Glucocorticoid
HSPA1B; HSPA1A	TC0600007616.hg.1	Glucocorticoid

© 2024 Boyle J et al. *JAMA Network Open.*

HSPA4	TC0500008664.hg.1	Glucocorticoid
HTR1A	TC0500010910.hg.1	Serotonin
HTR1B	TC0600012345.hg.1	Serotonin
HTR1D	TC0100013272.hg.1	Serotonin
HTR1E	TC0600008716.hg.1	Serotonin
HTR1F	TC0300008012.hg.1	Serotonin
HTR2A	TC1300008858.hg.1	Serotonin
HTR2B	TC0200016006.hg.1	Serotonin
HTR2C	TC0X00008178.hg.1	Serotonin
HTR3A	TC1100009098.hg.1	Serotonin
HTR3B	TC1100009097.hg.1	Serotonin
HTR3C	TC0300009686.hg.1	Serotonin
HTR3D	TC0300009685.hg.1	Serotonin
HTR3E	TC0300009688.hg.1	Serotonin
HTR4	TC0500012405.hg.1	Serotonin
HTR5A	TC0700009796.hg.1	Serotonin
HTR6	TC0100007175.hg.1	Serotonin
HTR7	TC1000011398.hg.1	Serotonin
NR3C1	TC0500012340.hg.1	Glucocorticoid
POU2F1	TC0100010499.hg.1	Glucocorticoid
POU2F2	TC1900010789.hg.1	Glucocorticoid
PTGES3	TC1200010931.hg.1	Glucocorticoid
SMAD3	TC1500007633.hg.1	Glucocorticoid
SMAD4	TC1800009229.hg.1	Glucocorticoid

Abbreviations: CTRA, Conserved Transcriptional Response to Adversity; IFN, interferon; TC, transcript cluster ID
‡I*FI30* is the gene that is listed in the CTRA
^*CXCL8* is synonymous with *IL8*; *IFIT1B* is synonymous with *IFIT1L*, and *IGLL3P* is synonymous with *IGLL3* in the CTRA

© 2024 Boyle J et al. *JAMA Network Open.*

eTable 2. Summary of Comparing Socioeconomic and Health Care Access Indicators Between Counties and Census Tracts in Maryland Contributing vs Not Contributing Participants to our Sample of Men Who Received Radical Prostatectomy Surgery for Prostate Cancer at the University of Maryland Medical Center, 1992-2021

Domain	Characteristic	P-value
Education	% Adults in county with less than high school diploma, 2017-2021	.38
	% Adults in county with bachelor's degree or higher, 2017-2021	.74
Economics	County unemployment rate, 2010	.56
	County unemployment rate, 2021	.59
	County median household income, 2021	.69
	% in county in poverty, 2021	.38
Healthcare Access	Less/Greater than 30 miles from census tract centroid to closest NCI designated cancer center	.08
	Less/Greater than 40 miles from census tract centroid to closest NCI designated cancer center	.20

Note: County-level data derived from United States Department of Agriculture Economic Research Service datasets, 2017-2021[1]. Access to healthcare defined by minimum distance to closest National Cancer Institute designated cancer center[2]. Socioeconomic indicators tested with two-sample unequal variance t-tests and healthcare access tested with chi-squared tests of independence.

© 2024 Boyle J et al. *JAMA Network Open.*

eTable 3. Summary of Genes Associated With the Neighborhood Measures in Adjusted Models for African American Men Who Received Radical Prostatectomy Surgery for Prostate Cancer at the University of Maryland Medical Center, 1992-2021

Measure	TC ID	Gene	Beta (se)[a]	p[a]	q[b]
	TC0100007175.hg.1	*HTR6*	0.003 (0.001)	.002	.25
	TC1100008123.hg.1	*ADRBK1*	0.003 (0.001)	.01	.35
	TC0500012405.hg.1	*HTR4*	0.004 (0.001)	.01	.35
	TC1000008396.hg.1	*IFIT2*	0.005 (0.002)	.01	.35
	TC2100007205.hg.1	*MX2*	0.005 (0.002)	.02	.44
	TC0200013916.hg.1	*IL1B*	0.005 (0.002)	.03	.44
	TC2200008174.hg.1	*IGLL1*	0.003 (0.001)	.03	.44
	TC0400007836.hg.1	*CXCL8*	0.007 (0.004)	.04	.49
ADI	TC0100008815.hg.1	*IFI44L*	0.006 (0.003)	.04	.49
	TC0100007175.hg.1	*HTR6*	0.206 (0.064)	.002	.19
	TC0900008660.hg.1	*PTGS1*	0.418 (0.167)	.01	.44
	TC1200008920.hg.1	*OAS3*	0.271 (0.110)	.02	.44
	TC2200008174.hg.1	*IGLL1*	0.231 (0.101)	.02	.44
	TC0300009686.hg.1	*HTR3C*	0.315 (0.139)	.03	.44
	TC0500012405.hg.1	*HTR4*	0.262 (0.117)	.03	.44
	TC1000008396.hg.1	*IFIT2*	0.375 (0.169)	.03	.44
RI	TC1100008123.hg.1	*ADRBK1*	0.212 (0.101)	.04	.51
	TC0900008660.hg.1	*PTGS1*	0.346 (0.134)	.01	.74
	TC2100007205.hg.1	*MX2*	0.282 (0.121)	.02	.74
	TC1200008920.hg.1	*OAS3*	0.185 (0.084)	.03	.74
	TC0100018367.hg.1	*CHRM3*	0.325 (0.152)	.03	.74
	TC1000008396.hg.1	*IFIT2*	0.251 (0.122)	.04	.74
Redlining	TC1000008968.hg.1	*ADRB1*	-0.344 (0.168)	.04	.74
**Measure**	**TC ID**	**Gene**	**Beta[a]**	**(2.5th , 97.5th) P[a]**	**EP[a,c]**

© 2024 Boyle J et al. *JAMA Network Open.*

NDI	TC0100010244.hg.1	*IFI16*	0.388	(0.132, 0.682)	99.6%

Abbreviations: ADI, Area Deprivation Index; EP, exceedance probability; NDI, Neighborhood Deprivation Index; RI, Racial Isolation Index; se, standard error; TC; transcript cluster ID; 2.5th P and 97.5th P, boundaries of 95% credible interval for the beta coefficient

[a]From linear regression modeling the given gene in relation to the neighborhood measure, adjusted for age at surgery and year of surgery

[b]p-value adjusted for multiple comparisons using the Benjamini-Hochberg False Discovery Rate approach

[c]exceedance probability (probability of being greater than zero)

© 2024 Boyle J et al. *JAMA Network Open.*

eTable 4. Summary of Sensitivity Analysis Additionally Adjusting for Primary Payer at Diagnosis for Genes Associated With the Neighborhood Measures in Adjusted Models Among African American and White Men Who Received Radical Prostatectomy Surgery for Prostate Cancer at the University of Maryland Medical Center, 1992-2021

Measure	TC ID	Gene	Pathway	Beta[a]	p[a]	q[b]	% Change[c]
ADI (n=160)	TC0100007175.hg.1	*HTR6*	Serotonin	0.003	<.001	.01	1.6
	TC0500012405.hg.1	*HTR4*	Serotonin	0.004	.003	.15	21.4
	TC0300009685.hg.1	*HTR3D*	Serotonin	0.003	.006	.18	20.0
	TC1000008401.hg.1	*IFIT5*	Type I IFN responses	0.005	.009	.18	14.6
	TC1000008396.hg.1	*IFIT2*	Type I IFN responses	0.005	.009	.18	22.1
	TC0400007836.hg.1	*CXCL8*	Proinflammatory	0.008	.01	.21	2.1
	TC0200007096.hg.1	*FOSL2*	Proinflammatory	0.004	.02	.30	4.7
	TC0200013916.hg.1	*IL1B*	Proinflammatory	0.005	.02	.30	5.6
	TC2100007205.hg.1	*MX2*	Type I IFN responses	0.004	.03	.33	17.7
	TC0100008815.hg.1	*IFI44L*	Type I IFN responses	0.005	.04	.39	3.4
	TC1400007706.hg.1	*FOS*	Proinflammatory	0.010	.05	.43	12.0
RI (n=136)	TC0100007175.hg.1	*HTR6*	Serotonin	0.179	.005	.54	6.4
	TC2200008174.hg.1	*IGLL1*	Antibody synthesis	0.231	.02	.63	14.7
	TC0300009686.hg.1	*HTR3C*	Serotonin	0.324	.03	.63	12.1
	TC1000008396.hg.1	*IFIT2*	Type I IFN responses	0.342	.03	.63	10.2
	TC2100007205.hg.1	*MX2*	Type I IFN responses	0.318	.04	.63	15.4
	TC1100008123.hg.1	*ADRBK1*	Adrenergic	0.196	.04	.63	23.7
	TC1600008712.hg.1	*IRF8*	Type I IFN responses	0.327	.04	.63	6.7
Redlining (n=218)	TC0900008660.hg.1	*PTGS1*	Proinflammatory	0.337	.01	.59	0.4
	TC2100007205.hg.1	*MX2*	Type I IFN responses	0.288	.01	.59	8.1
	TC1000008396.hg.1	*IFIT2*	Type I IFN responses	0.266	.02	.59	18.6
	TC1000008968.hg.1	*ADRB1*	Adrenergic	-0.352	.02	.59	13.9
	TC0100018367.hg.1	*CHRM3*	Muscarinic	0.332	.03	.59	2.0
	TC1200008920.hg.1	*OAS3*	Type I IFN responses	0.185	.03	.59	6.0
**Measure**	**TC ID**	**Gene**	**Pathway**	**Beta[a]**	**(2.5th , 97.5th) P[a]**	**EP[a,d]**	**% Change[c]**
NDI (n=211)	TC1400007706.hg.1	*FOS*	Proinflammatory	0.195	(-0.045, 0.404)	95.8%	34.2

Abbreviations: ADI, Area Deprivation Index; EP, exceedance probability; IFN, interferon; NDI, Neighborhood Deprivation Index; RI, Racial Isolation Index; se, standard error; TC; transcript cluster ID; 2.5th P and 97.5th P, boundaries of 95% credible interval for the beta coefficient

[a]From linear regression modeling the given gene in relation to the neighborhood measure, adjusted for race, age at surgery, year of surgery, and primary payer at diagnosis

© 2024 Boyle J et al. *JAMA Network Open.*

[b]p-value adjusted for multiple comparisons using the Benjamini-Hochberg False Discovery Rate approach

[c]Percent change (absolute) in beta when conducting sensitivity analysis that additionally adjusted models for primary payer at diagnosis (private insurance, Medicaid, Medicare, Military/Veterans Affairs, unspecified insurance, uninsured)

[d]exceedance probability (probability of being greater than zero)

© 2024 Boyle J et al. *JAMA Network Open.*

eFigure 1. Flowchart Illustrating Sample Selection

*denotes oversampling of African American men.

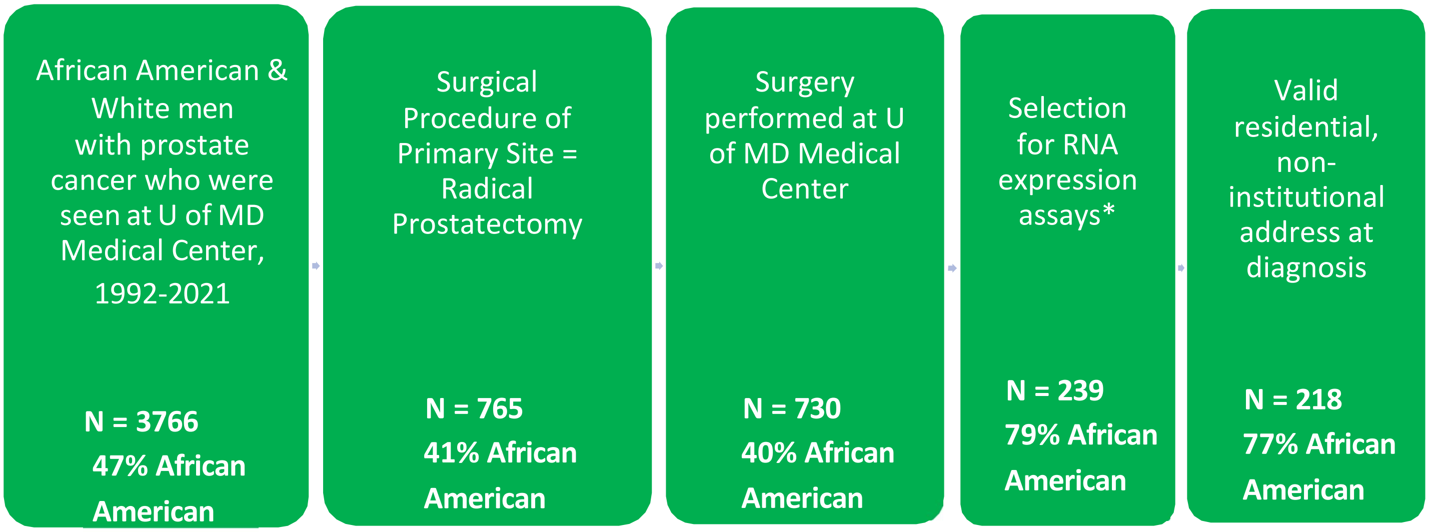

© 2024 Boyle J et al. *JAMA Network Open.*

eFigure 2. Area Deprivation Index Values of Participants in Sample

Higher values indicate greater neighborhood deprivation. Participant locations are slightly jittered to maintain confidentiality.

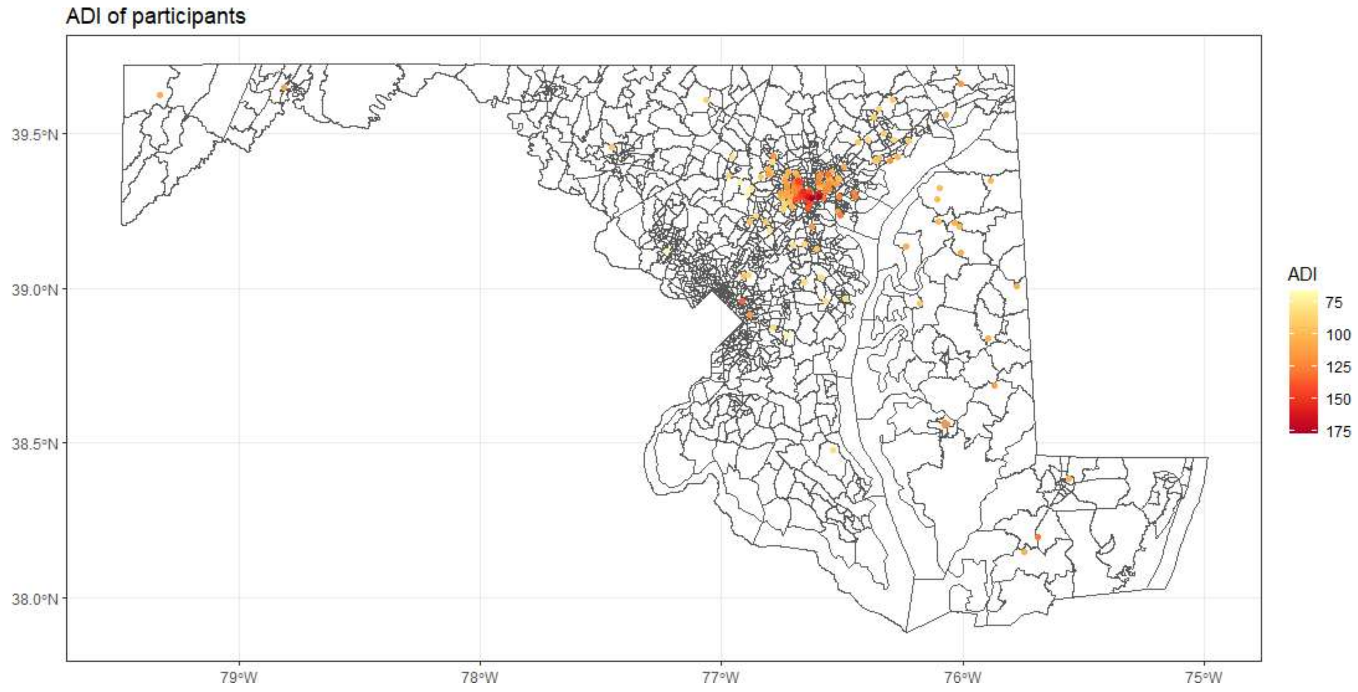

© 2024 Boyle J et al. *JAMA Network Open.*

eFigure 3. Racial Isolation Index Values of Participants in Sample

Higher values indicate greater African American residential segregation. Participant locations are slightly jittered to maintain confidentiality.

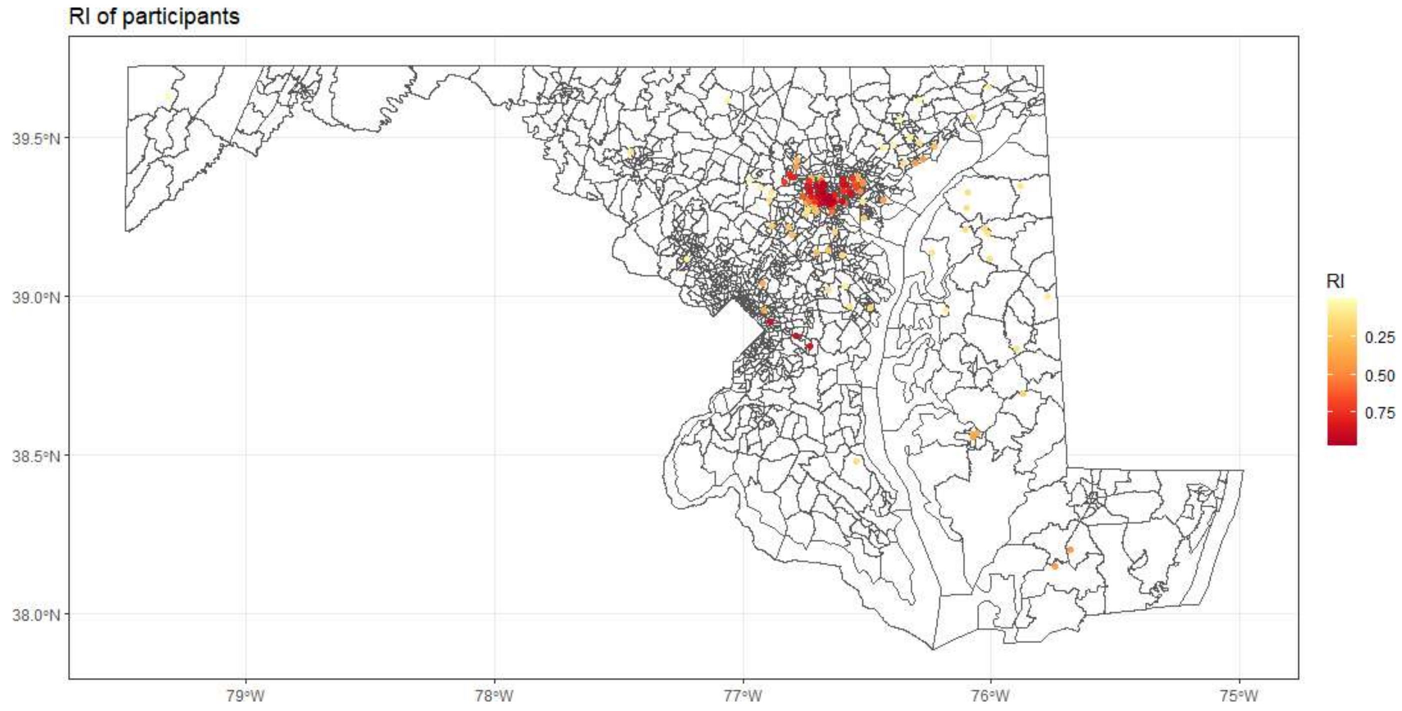

© 2024 Boyle J et al. *JAMA Network Open.*

eMethods.

Risk Stratification

We categorized the participants in our sample into prostate cancer risk stratification categories according to guidelines set by the American Urological Association (AUA)[3]. Specifically, we used information on grade group, tumor stage, and pre-operative prostate-specific antigen (PSA) to classify participants as low-risk, intermediate-risk, or high-risk. We used pathologic Gleason score to determine the grade group and pathologic tumor stage to determine the tumor stage since the study participants comprised men who received radical prostatectomy surgery for prostate cancer. In the case that participants satisfied two of the three criteria for low-risk prostate cancer according to AUA guidelines (PSA < 10 ng/mL, grade group 1, and stage 1-2A) but had a missing value for the other criterion, we considered such participants to have low-risk prostate cancer.

RNA expression data

We leveraged available transcriptomic data from formalin-fixed, paraffin-embedded (FFPE) prostate tumor tissue from our previous studies. The pathology and RNA extraction approaches have been described previously[4]. Briefly, pathology review identified the most representative tumor sample from the FFPE blocks. Based on an H&E slide, we circled ~2 mm tumor regions (>=75% purity), then cut two unstained double-thickness slides (10 µm) per sample, with the same circled areas marked for RNA extraction. RNA was extracted using Qiagen's RNEasy FFPE kit. We obtained RNA expression data from the Human Clariom D™ array, which measures expression for 138,745 transcript cluster (TC) IDs. We analyzed a subset of 105 TCs corresponding to stress-related genes, including those in the Conserved Transcriptional Response to Adversity (CTRA)[5] and stress-related signaling pathways, encompassing the adrenergic, glucocorticoid, dopaminergic, serotoninergic, and muscarinic systems[6] (eTable 1). Various stress-related signaling genes have been previously linked with lethal prostate cancer[6] and there is relevance of CTRA genes (e.g., proinflammatory genes) to prostate cancer risk and progression[7–9]. Similar to our previous work[4], we performed SST-RMA normalization and log base-2 transformation of the RNA expression data with TAC software, version 4.0.

Neighborhood variables

We included several measures of neighborhood disadvantage in the present study, considering the census tract containing each participant's address at diagnosis to constitute the neighborhood. The first such measure was the Area Deprivation Index (ADI)[10,11], which includes 17 neighborhood-level variables in the income, education, employment and housing quality domains and employs 5-year estimates from the American Community Survey (ACS) beginning in 2005. This index uses a set of fixed importance weights for component variables that were estimated in a previous factor analysis[11] that do not consider associations with the present outcome. The ADI has been used in several previous analyses of neighborhood deprivation[12–14] and is standardized to have a mean score of 100 and standard deviation of 20, with greater values indicating greater deprivation. We used the R package "sociome" to obtain ADI scores. We calculated the ADI measure for 160 participants (117 African American and 43 White participants).

Our second measure was a custom neighborhood deprivation index (NDI) that we fit with the validated Bayesian index model developed by our group[15]. The construction of the NDI was similar to that of the ADI, but importance weights for components in the index were estimated in modeling, allowing identification of the most important variables in the index. We have found in previous studies that the Bayesian index model is able to accurately and consistently estimate index effects in addition to the relative importance of component variables in the index[15] and that an NDI can provide better model fit than other measures that assume equal importance for all components in the index[16]. We included 16 census tract-level variables in the NDI that we have used in previous work[17,18]: median household income, median house value, percent of structures built in 1939 or earlier, median rent as a percentage of household income, percent renters, percent in poverty, percent receiving public assistance/SNAP benefits, percent vacant structures, percent Black, percent Hispanic, percent without high school diploma, percent unemployed, percent single-parent households with children under 18, percent with no vehicle, percent lacking complete plumbing facilities, and percent crowded households. While many variables included in the ADI and NDI captured common themes (i.e., house value, income, education, and children), there were a few differences; the ADI included percent in white-collar occupations and income disparity, and the NDI included more racial and housing

© 2024 Boyle J et al. *JAMA Network Open.*

characteristics. We inverted the household income and house value variables in the NDI so that all variables would be in the direction of greater deprivation and converted these variables to all be in 2020 inflation-adjusted U.S. dollars. We calculated the NDI measure for 211 participants (162 African American and 49 White participants).

The third measure we used was the spatial Racial Isolation (RI) index[19], which was available for 2009 and later. This measure ranges from 0 to 1 for a given racial group and considers the racial composition of census geographies as well as that of their neighbors. Following previous research, we calculated the racial isolation of African American residents, conceptualizing increasing values of this measure to proxy increasing concentrations of social and economic disadvantage, in response to the continuing history of anti-Black racism in the United States[20–23]. We calculated the RI measure for 136 participants (99 African American and 37 White participants).

We considered historical redlining for our final neighborhood measure given the associations identified in the literature between living in a formerly redlined neighborhood and negative health outcomes. The process of redlining occurred when the Home Owners' Loan Corporation (HOLC) assigned "grades" to neighborhoods in urban areas. These grades were a proxy of HOLC's perceived risk for real estate investment in these areas, and were often a function of neighborhood racial, ethnic, and/or religious characteristics[24]. Redlining influenced the likelihood of HOLC providing mortgages, as well as refinancing (to avoid foreclosure on existing mortgages), for individuals in the neighborhood. Living in historically redlined neighborhoods has been associated with a variety of negative health outcomes in the present day[25]. We used the HOLC redlining map of Baltimore from the 1930s, which overlaid neighborhood grades of A, B, C, and D on George Cram's 1935 "Street Map of Baltimore Area" that also included Towson, Pikesville, and Catonsville. Certain neighborhoods, such as those outside of the city center, did not receive a grade from HOLC. For this measure we considered the exposure to be binary, taking on a value of 1 if the residence fell within a neighborhood graded D (the worst grade) on the HOLC map, and 0 otherwise (A, B, C, or no grade). We used the digitized redlining map produced by the Mapping Inequality project (University of Richmond)[26]. We calculated the redlining measure for 218 participants (168 African American and 50 White participants).

Statistical analysis

We fit linear regression models for each gene in relation to each neighborhood measure, adjusting for race (African American and White), age at surgery (<=49, 50-59, 60-69, and 70+ years), and year of surgery (1992-1997, 1998-2004, 2005-2010, 2011-2016, and 2017-2021, with groupings defined based on our study distribution). For the ADI, RI, and historical redlining exposures, we fit frequentist linear regression models with the log base-2 transformed gene expression values as the response. We reported coefficients, standard errors, and p-values, as well as q-values (p-values adjusted for the 105 genes tested with each exposure with the Benjamini- Hochberg method[27]), using a conventional .05 level of significance. For the NDI, we fit Bayesian linear regression models, reporting coefficients, credible intervals, and exceedance probabilities (EPs), considering EPs of 90% or greater to constitute significance. We used the R package "rjags" for model fitting[28]. In the Bayesian models we assigned vague Normal priors to each regression coefficient, $\beta_i \sim Normal(0, \tau_i), \tau_i = 1/\sigma^2, \sigma_i \sim Unif(0,10)$. Additionally, we assigned a Dirichlet prior with vector of parameters $\boldsymbol{\alpha}$ for the index weights in the NDI so that each weight would be between 0 and 1 and the weights would sum to 1. We also calculated the Spearman correlations between the continuous neighborhood measures to evaluate their agreement. As a sensitivity analysis for the associations between the neighborhood measures and the stress-related genes, we repeated the models among African American men only to assess whether the significant findings were similar to the primary analysis among African American and White men combined, adjusting for age and year of surgery. We also assessed characteristics of the larger group of African American and White men receiving radical prostatectomy for prostate cancer at the University of Maryland Medical Center from 1992-2021 to assess the extent to which our sample reflected this larger group. We performed an additional sensitivity analysis that compared socioeconomic and healthcare access indicators[1,2] of geographic areas in Maryland (census tracts, counties) contributing to our sample vs. those that did not contribute to explore the representativeness of our sample with respect to Maryland as a whole. Finally, we assessed whether primary payer at diagnosis, which would be associated with individual-level socioeconomic status, was a confounder of our top associations (those with p<.05 in our primary analysis) in another sensitivity analysis. We performed all analyses in R, version 4.3.1[29]; tests were two-tailed.

© 2024 Boyle J et al. *JAMA Network Open.*

eReferences.

1. US Department of Agriculture. County-level Data Sets. Economic Research Service - U.S. Department of Agriculture. Accessed December 14, 2023. https://www.ers.usda.gov/data-products/county-level-data-sets/

2. National Cancer Institute. Catchment Areas of NCI-Designated Cancer Centers. Accessed December 14, 2023. https://gis.cancer.gov/ncicatchment/app/

3. American Urological Association. Clinically Localized Prostate Cancer: AUA/ASTRO Guideline (2022). Accessed April 22, 2024. https://www.auanet.org/guidelines-and-quality/guidelines/clinically-localized-prostate-cancer-aua/astro-guideline-2022

4. Barry KH, Mohanty K, Erickson PA, et al. MYC DNA methylation in prostate tumor tissue is associated with Gleason score. *Genes*. 2020;12(1):12.

5. Fredrickson BL, Grewen KM, Coffey KA, et al. A functional genomic perspective on human well-being. *Proc Natl Acad Sci U S A*. 2013;110(33):13684-13689. doi:10.1073/pnas.1305419110

6. Lu D, Sinnott JA, Valdimarsdóttir U, et al. Stress-Related Signaling Pathways in Lethal and Nonlethal Prostate Cancer. *Clin Cancer Res*. 2016;22(3):765-772. doi:10.1158/1078-0432.CCR-15-0101

7. Rani A, Dasgupta P, Murphy JJ. Prostate Cancer: The Role of Inflammation and Chemokines. *Am J Pathol*. 2019;189(11):2119-2137. doi:10.1016/j.ajpath.2019.07.007

8. Platz EA, Kulac I, Barber JR, et al. A prospective study of chronic inflammation in benign prostate tissue and risk of prostate cancer: linked PCPT and SELECT cohorts. *Cancer Epidemiology, Biomarkers & Prevention*. 2017;26(10):1549-1557.

9. Zhang Y, Zhou CK, Rencsok EM, et al. A prospective study of intraprostatic inflammation, focal atrophy, and progression to lethal prostate cancer. *Cancer Epidemiology, Biomarkers & Prevention*. 2019;28(12):2047-2054.

10. Kind AJH, Jencks S, Brock J, et al. Neighborhood socioeconomic disadvantage and 30-day rehospitalization: a retrospective cohort study. *Ann Intern Med*. 2014;161(11):765-774. doi:10.7326/M13-2946

11. Singh GK. Area deprivation and widening inequalities in US mortality, 1969-1998. *Am J Public Health*. 2003;93(7):1137-1143. doi:10.2105/ajph.93.7.1137

12. Hu J, Kind AJ, Nerenz D. Area deprivation index predicts readmission risk at an urban teaching hospital. *American Journal of Medical Quality*. 2018;33(5):493-501.

13. KC M, Oral E, Straif-Bourgeois S, Rung AL, Peters ES. The effect of area deprivation on COVID-19 risk in Louisiana. *Plos one*. 2020;15(12):e0243028.

14. Yu KX, Yuan WJ, Huang CH, et al. Socioeconomic deprivation and survival outcomes in patients with colorectal cancer. *American Journal of Cancer Research*. 2022;12(2):829.

© 2024 Boyle J et al. *JAMA Network Open.*

15. Wheeler DC, Rustom S, Carli M, Whitehead TP, Ward MH, Metayer C. Bayesian Group Index Regression for Modeling Chemical Mixtures and Cancer Risk. *International journal of environmental research and public health*. 2021;18(7):3486.

16. Wheeler DC, Jones RM, Schootman M, Nelson EJ. Explaining variation in elevated blood lead levels among children in Minnesota using neighborhood socioeconomic variables. *Science of the Total Environment*. 2019;650:970-977. doi:10.1016/j.scitotenv.2018.09.088

17. Boyle J, Ward MH, Cerhan JR, Rothman N, Wheeler DC. Modeling historic neighborhood deprivation and non-Hodgkin lymphoma risk. *Environmental Research*. Published online 2023:116361.

18. Wheeler DC, Boyle J, Barsell DJ, et al. Neighborhood deprivation is associated with increased risk of prenatal smoke exposure. *Prevention Science*. Published online 2022:1-12. doi:https://doi.org/10.1007/s11121-022-01355-7

19. Anthopolos R, James SA, Gelfand AE, Miranda ML. A spatial measure of neighborhood level racial isolation applied to low birthweight, preterm birth, and birthweight in North Carolina. *Spatial and Spatio-temporal Epidemiology*. 2011;2(4):235-246. doi:10.1016/j.sste.2011.06.002

20. Kong AY, Herbert L, Feldman JM, Trangenstein PJ, Fakunle DO, Lee JGL. Tobacco and Alcohol Retailer Availability and Neighborhood Racialized, Economic, and Racialized Economic Segregation in North Carolina. *Journal of Racial and Ethnic Health Disparities*. Published online 2022:1-11.

21. Jung MK, Vargas JC, Bonilla-Silva E. *State of White Supremacy: Racism, Governance, and the United States*. Stanford University Press; 2011.

22. Bonilla-Silva E. *White Supremacy and Racism in the Post-Civil Rights Era*. Lynne Rienner Publishers; 2001.

23. Miranda ML, Maxson P, Edwards S. Environmental contributions to disparities in pregnancy outcomes. *Epidemiologic reviews*. 2009;31(1):67-83.

24. Squires GD, Woodruff F. Redlining. In: *The Wiley Blackwell Encyclopedia of Urban and Regional Studies*. ; 2019:1-8. doi:10.1002/9781118568446.eurs0260

25. Swope CB, Hernández D, Cushing LJ. The Relationship of Historical Redlining with Present-Day Neighborhood Environmental and Health Outcomes: A Scoping Review and Conceptual Model. *Journal of Urban Health*. 2022;99(6):959-983. doi:10.1007/s11524-022-00665-z

26. Digital Scholarship Lab, University of Richmond. Mapping Inequality: Redlining in New Deal America. Published June 23, 2023. Accessed July 23, 2023. https://dsl.richmond.edu/panorama/redlining/

© 2024 Boyle J et al. *JAMA Network Open.*

27. Benjamini Y, Hochberg Y. Controlling the false discovery rate: a practical and powerful approach to multiple testing. *Journal of the Royal statistical society: series B (Methodological)*. 1995;57(1):289-300.

28. Plummer M, others. JAGS: A program for analysis of Bayesian graphical models using Gibbs sampling. In: *Proceedings of the 3rd International Workshop on Distributed Statistical Computing*. Vol 124. Vienna, Austria.; 2003:1-10.

29. R Core Team. R: A language and environment for statistical computing. Published online 2021.

© 2024 Boyle J et al. *JAMA Network Open.*
