# Supplementary material for: Neighborhood Disadvantage and Prostate Tumor RNA Expression of Stress-Related Genes
Source: JAMA Netw Open. 2024 Jul 12;7(7):e2421903. doi: 10.1001/jamanetworkopen.2024.21903 (PMC11245728; doi:10.1001/jamanetworkopen.2024.21903)
Supplement: Supplement 2. — Data Sharing Statement [file jamanetwopen-e2421903-s002.pdf]

Data Sharing Statement

Boyle. Neighborhood Disadvantage and Prostate Tumor RNA Expression of Stress-Related Genes. *JAMA Netw Open*. Published July 12, 2024.
doi:10.1001/jamanetworkopen.2024.21903

Data
Data available: Yes
Data types: Deidentified participant data
How to access data: The data presented in this paper will be made available in the Gene Expression Omnibus (GEO) database, without patient identifiers.
When available: With publication

Supporting Documents
Document types: None

Additional Information
Who can access the data: The individual-level data for this project will be made available through controlled-access. Use of the data must be related to the specified disease (prostate cancer). Additional requirements include publication (requestor agrees to make results of studies using the data available to the larger scientific community) and collaboration [requestor must provide a letter of collaboration with the primary study investigator(s)].
Types of analyses: Use of the data must be related to the specified disease (prostate cancer). Allowed uses of the data include methods development research (e.g., development of software or algorithms).
Mechanisms of data availability: The data will be made available after approval of the proposal by the primary study investigator(s) and with a signed data access agreement.
